# Supra- and infra-tentorial degeneration patterns in primary lateral sclerosis: a multimodal longitudinal neuroradiology study

**DOI:** 10.1007/s00415-024-12261-z

**Published:** 2024-03-05

**Authors:** Jana Kleinerova, Marlene Tahedl, Ee Ling Tan, Siobhan Delaney, Jennifer C. Hengeveld, Mark A. Doherty, Russell L. McLaughlin, Orla Hardiman, Kai Ming Chang, Eoin Finegan, Peter Bede

**Affiliations:** 1 Computational Neuroimaging Group (CNG), School of Medicine, Trinity College Dublin, Dublin 2, Ireland; 2 Department of Neurology, St James’s Hospital, Dublin, Ireland; 3 Smurfit Institute of Genetics, Trinity College Dublin, Dublin, Ireland

**Keywords:** Primary Lateral Sclerosis, Cerebellum, Connectivity, Pharmaceutical trials, MRI

## Abstract

**Background:**

Primary lateral sclerosis (PLS) is traditionally solely associated with progressive upper motor neuron dysfunction manifesting in limb spasticity, gait impairment, bulbar symptoms and pseudobulbar affect. Recent studies have described frontotemporal dysfunction in some patients resulting in cognitive manifestations. Cerebellar pathology is much less well characterised despite sporadic reports of cerebellar disease.

**Methods:**

A multi-timepoint, longitudinal neuroimaging study was conducted to characterise the evolution of both intra-cerebellar disease burden and cerebro-cerebellar connectivity. The volumes of deep cerebellar nuclei, cerebellar cortical volumes, cerebro-cerebellar structural and functional connectivity were assessed longitudinally in a cohort of 43 individuals with PLS.

**Results:**

Cerebello-frontal, -temporal, -parietal, -occipital and cerebello-thalamic structural disconnection was detected at baseline based on radial diffusivity (RD) and cerebello-frontal decoupling was also evident based on fractional anisotropy (FA) alterations. Functional connectivity changes were also detected in cerebello-frontal, parietal and occipital projections. Volume reductions were identified in the vermis, anterior lobe, posterior lobe, and crura. Among the deep cerebellar nuclei, the dorsal dentate was atrophic. Longitudinal follow-up did not capture statistically significant progressive changes. Significant primary motor cortex atrophy and inter-hemispheric transcallosal degeneration were also captured.

**Conclusions:**

PLS is not only associated with upper motor neuron dysfunction, but cerebellar cortical volume loss and deep cerebellar nuclear atrophy can also be readily detected. In addition to intra-cerebellar disease burden, cerebro-cerebellar connectivity alterations also take place. Our data add to the evolving evidence of widespread neurodegeneration in PLS beyond the primary motor regions. Cerebellar dysfunction in PLS is likely to exacerbate bulbar, gait and dexterity impairment and contribute to pseudobulbar affect.

**Supplementary Information:**

The online version contains supplementary material available at 10.1007/s00415-024-12261-z.

## Introduction

PLS has traditionally been exclusively associated with primary motor cortex, corpus callosum and descending corticospinal tract degeneration [1–4], but there is a scarcity of longitudinal studies in PLS. [5–7]. There is however growing evidence of frontotemporal disease burden and supporting clinical evidence of frontotemporal dysfunction [8–12]. More recently, considerable subcortical disease burden has also been described, and putative extrapyramidal and cognitive correlates proposed [13–15]. There is a particularly high incidence of pseudobulbar affect in PLS, which is classically linked to cortico-bulbar disconnection [16, 17], but the contribution of impaired cerebellar gating is increasingly recognised as an important aetiological factor [18–23]. As PLS carries a considerably better prognosis than ALS [24], it is often inaccurately regarded as a relatively benign condition. However, recent research has helped to reconceptualise PLS from a pure UMN condition with a relatively benign course, to a relentlessly progressive neurodegenerative condition [25] with considerable extra-motor, frontotemporal, subcortical and cerebellar involvement. Despite landmark histopathology papers [26], the post-mortem literature on PLS is relatively scarce, therefore quantitative imaging approaches are the most reliable techniques to characterise and track progressive disease burden trajectories. Both PET and MRI studies have contributed considerable insights, but the majority of imaging studies are cross-sectional. Previous diagnostic criteria required a minimum symptom duration of 4 years, which led to the inclusion of patients with considerable disease durations into research studies. The new consensus diagnostic criteria [27] and the introduction of the category “probable” PLS have enabled earlier recruitment and enrolment into academic research studies [28]. The characterisation of cerebellar pathology is a relatively novel frontier of PLS research, particularly in view of conflicting histopathology reports and the difficulty to appreciate clinical correlates of cerebellar disease. Cerebellar manifestations are likely to be masked by the predominant upper motor neuron signs and admittedly formal cerebellar assessments are not commonly performed in PLS. Some post-mortem reports described the cerebellum as ‘unremarkable’ [29] despite radiological reports of cerebellar involvement [9, 30, 31]. Global cerebellar volume loss is sometimes reported, but the predilection for specific cerebellar lobules is less well evaluated [32]. Recent quantitative imaging studies confirmed functional [33, 34] and structural cerebro-cerebellar connectivity changes [33, 34] and spinocerebellar tract degeneration have also been recently reported. [35] Considerable brainstem atrophy has also been shown in PLS, based on high-resolution structural data, which may be exacerbated by the degeneration of cerebellar projections. [36, 37] Notwithstanding the radiological evidence, overt cerebellar ataxia is not commonly noted clinically observed in PLS. [34, 35, 38]. Cerebellar pathology in ALS is better characterised [39], in no small part due to the interest in *ATXN1* and *ATXN2* repeat expansions, and genotype-associated cerebellar signatures have been proposed [40]. Evidence from other neurodegenerative conditions suggests that cerebellar pathology is likely to contribute to bulbar, eye-movement, gait, dexterity, cognitive, behavioural, pseudobulbar and respiratory manifestations in PLS also [38, 41–48]. Analogous to ALS [49–51], language deficits, behavioural impairment, deficits in social cognition and executive dysfunction have also been reported in PLS [8–12]. In view of recent imaging and clinical reports, our objective was the systematic characterisation of both intra-cerebellar and cerebro-cerebellar connectivity alterations in a large cohort of individuals with PLS and track integrity measures longitudinally over four timepoints.

## Methods

### Ethics approval

This project was approved by the Ethics Committee of Beaumont Hospital Dublin (REC reference: 08/90) and each participant gave informed consent prior to study enrolment.

### Demographic, clinical and genetic profiling

A total of 156 participants, 43 PLS patients and 113 healthy controls (HC) were included in this study. Up to four MRI scans were acquired with a follow-up interval of four months. Demographic variables were carefully recorded; age, sex, symptom duration, handedness, education, and family history of neurodegenerative conditions (Table 1). Forty out of 43 patients had their total ALSFRS-r, ALSFRS sub-scores, symptom duration, Penn Upper Motor Neuron Score (PUMNS), modified Ashworth spasticity scale scores, Edinburgh Cognitive and Behavioural ALS Screen (ECAS), the Frontal Systems Behavior Scale (FrSBe), the Hospital Anxiety and Depression Scale (HADS), the Emotional Lability Questionnaire (ELQ) and the Center for Neurological Study-Lability Scale (CNS-LS) scores also recorded. A standardised cerebellar assessment was also performed on 31 patients by the same neurologist. Individuals with PLS were diagnosed based on the new consensus criteria [27]. Exclusion criteria for each participant included prior stroke, brain or neurovascular surgery, traumatic brain injury, and comorbid neurological or psychiatric diagnoses. Thirty-one of the 43 PLS patients underwent whole genome sequencing as described previously [52] and were screened for ALS [53] and HSP-associated [54] genetic variants. Thirty-three PLS were screened for *C9orf72* GGGGCC repeat expansions using repeat-primed polymerase chain reaction (PCR). GeneMapper version 4.0 was used to visualise capillary electrophoresis outcomes more than 30 repeats were considered *C9orf72*-positive.

**Table 1:** Demographic details of the study population

	PLS	HC	PLS vs HC t-test ^+^/Chi-square test^++^
Number of subjects at baseline (T1/dMRI/rs-fMRI)	43 (43/41/39)	113 (113/113/111)	*n.a*
Number of subjects at Time-point 2 (T1/ dMRI/rs-fMRI)	8 (30/29/27)	18 (18/18/18)	*n.a*
Number of subjects at Time-point 3 (T1/ dMRI/rs-fMRI)	7 (25/25/23)	13 (13/13/13)	*n.a*
Number of subjects at Time-point 4 (T1/ dMRI/rs-fMRI)	3 (20/19/15)	8 (8/8/8)	*n.a*
Age [y, mean ± SD]	55.50 ± 9.05	59.36 ± 10.66	*t*(80.33) = 1.33, *p* = .188
Years of education [y, mean ± SD]	12.18 ± 3.25	14.77 ± 3.46	*t*(71.04) = – 4.05, *p* = .001^*^
Sex, F/M	16/27	57/56	*X*^*2*^(1, *N* = 156) = 1.69, *p* = .193
Handedness, R/L	39/4	106/7	*X*^*2*^(1, *N* = 156) = 0.11, *p* = .736
Years of symptom duration [y, mean ± SD]	109.75 ± 70.46	*n.a*	*n.a*

^#^Follow-up scans were acquired with an inter-scan interval of four months

*dMRI* diffusion-weighted imaging, *F* female, *rs-fMRI* resting-state functional MRI, *HC* healthy control, *L* left-handed, *M* male, *MRI* magnetic resonance imaging, *n.a.* not applicable / not available, *PLS* primary lateral sclerosis, *R* right-handed, *SD* standard deviation, *y* years

^+^Welch two-sample t-tests [*t*] were performed to test differences of age and years of education between all PLS vs. HC

^++^Chi-square tests [*X*^*2*^] were performed to test differences of sex and handedness frequencies between all PLS patients vs. HC

^*^significant at an alpha-level of *p* ≤ 0.05

### Neuroimaging

A standardised neuroimaging protocol was implemented on a 3 Tesla Philips Achieva MR scanner. The protocol included T1-weighted (T1w), fluid-attenuated inversion recovery (FLAIR), diffusion-MRI (dMRI), resting state functional MRI (rs-fMRI) sequences. A 3D Inversion Recovery prepared Spoiled Gradient Recalled echo (IR-SPGR) sequence was implemented to acquire T1w images with: TR / TE = 8.5/3.9 ms, TI = 1060 ms, FOV of 256 × 256 × 160 mm, 160 sagittal slices with no interslice gap, flip angle (FA) = 8°, VR = 1 mm^3^, SENSE factor = 1.5. An Inversion Recovery Turbo Spin Echo (IR-TSE) sequence was utilised to acquire FLAIR images axially with a repetition time (TR) / echo time (TE) = 11,000/125 ms, inversion time (TI) = 2800 ms, field of view (FOV) = 230 × 183 × 150 mm, voxel resolution (VR) = 0.65 × 0.87 × 4 mm. A spin-echo echo planar imaging (SE-EPI) pulse-sequence was used to record dMRI data with a 32-direction Stejskal-Tanner diffusion encoding scheme: TR/TE = 7639/59 ms, FOV = 245 × 245 × 150 mm, 60 axial slices with no interslice gaps, FA = 90°, VR = 2.5 mm^3^, SENSE factor = 2.5, dynamic stabilisation and spectral presaturation with inversion recovery (SPIR) fat suppression. An echo-planar imaging (EPI) sequence was used to acquire 220 volumes of rs-fMRI data to assess blood oxygen level-dependent (BOLD) signal at rest with TR/TE = 2000/35 ms, FOV = 233 × 233 × 120 mm, 30 axial slices with no interslice gap, FA = 90°, VR = 2.875 mm × 2.875 mm × 4 mmm, SENSE factor = 2.5. Participants were instructed to close their eyes during the rs-fMRI data acquisition.

### Structural analyses

**Fig. 1:**
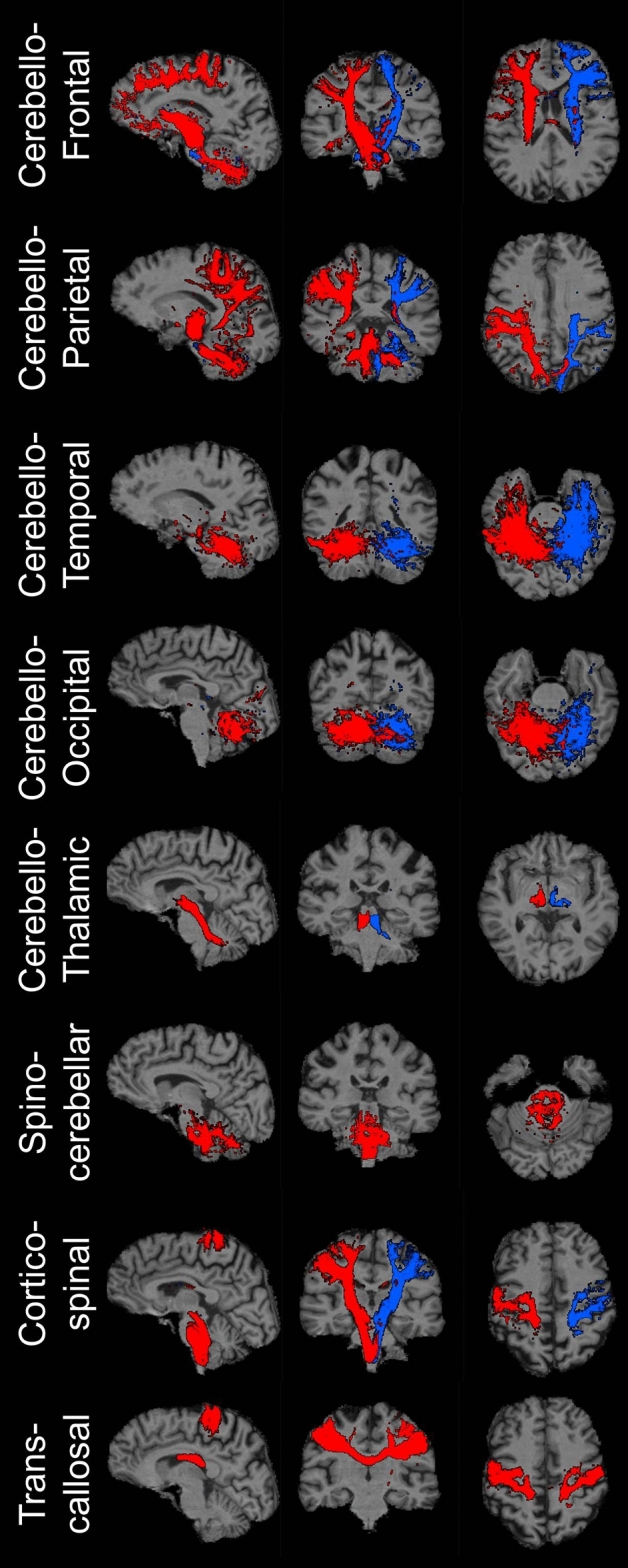
Illustrative tractography outputs from a healthy control. Right-hemispheric and unilateral tracts are depicted in red, left-hemispheric tracts in blue

The Computational Anatomy Toolbox (CAT12) [55] was implemented to assess cerebellar grey matter (GM) alterations. Pre-processing steps included denoising, affine registration, partial volume segmentation, skull-stripping and spatial normalization. Cerebellar cortical regions-of-interest (ROIs) were defined using the SUIT cerebellar segmentation pipeline [56]: (1) anterior lobe (SUIT labels I-V), (2) posterior lobe (SUIT labels VI-IX), (3) flocculonodular lobe (SUIT) label X, (4) crura (SUIT labels “Crura I” and “Crura II”), and (5) vermis (SUIT labels “Vermis”). The Julich-Brain Cytoarchitectonic Atlas was used to define the cerebellar nuclei [57]: (1) dorsal dentate, (2), ventral dentate, (3) interposed, and (4) fastigial. The primary motor cortex (M1) was defined based on the labels “Brodmann 4a” labels from the Anatomy3 atlas [58]. GM volume of these ROIs were estimated separately in the two hemispheres using CAT12 and added to generate a single volume output for each ROI. *MRtrix3* was utilised for dMRI data pre-processing [59], which included denoising, Gibb’s Ringing artifact removal, motion-, eddy current- and bias field-corrections. Subsequent to dMRI data preprocessing, the constrained spherical deconvolution (CSD) approach was implemented [60] to estimate fibre orientation distribution (fODF) in each voxel before normalisation [61]. Tractography of the following tracts was performed: (1) cerebello-frontal, (2) cerebello-parietal, (3) cerebello-temporal, (4), cerebello-occipital, (5) cerebello-thalamic and (6) spinocerebellar i.e. inferior brainstem-cerebellum. Relevant masks were defined using SUIT, Automated Anatomical Labeling (AAL) atlas and Hammers atlas labels [62]. The corticospinal tract was mapped from the primary motor cortex to the brainstem in each hemisphere separately and transcallosal fibres were mapped between the right to left motor cortex. Probabilistic tractography [63] was performed with 5000 streamlines and the Track Density Imaging (TDI) approach was implemented [64] (Fig. 1). Two white matter integrity metrics, fractional anisotropy (FA) and radial diffusivity (RD) were evaluated for each tract.

### Functional analyses

BOLD signal correlations were explored between the above ROIs, along the CST and CC. The FEAT pipeline of the FMRIB Software Library (FSL) [65] was used to pre-process fMRI data including brain extraction, intensity normalisation, and slice-time corrections. FSL’s ICA-based Automatic Removal Of Motion Artifacts (ICA-AROMA) [66] was utilised to correct for head-motion artifacts. Data were registered to MNI152 2mm standard space. As above, (1) cerebello-frontal, (2) cerebello-parietal, (3) cerebello-temporal, (4), cerebello-occipital, (5) cerebello-thalamic and (6) cerebello-spinal functional connectivity were appraised. Fisher *z*-transformed Pearson correlations were run between the mean BOLD time courses of the above pairs of ROIs in Matlab R2022b (The Mathworks, Natick, USA) using the CoSMoMVPA [67] toolbox.

### Statistical modelling

Statistical analyses were performed with RStudio (version 2022.12.0 + 353; R version 4.2.2).

Differences in means of age and education between PLS patients and HC were investigated using Welch two-sample t-tests, whereas differences in sex and handedness frequencies were compared using Chi-square testing. To test for cross-sectional differences in neuroimaging metrics between PLS patients and HC, we implemented a one-way analysis of variance (ANOVA), correcting for the confounding effects of age, sex, handedness, and years of education. In our volumetric analyses, we additionally corrected for total cerebellar volume for cerebellar GM analyses and total intracranial volume (TIV) for cortical GM analyses. We evaluated the main effect of the Group (i.e., PLS/HC). To test for longitudinal differences in neuroimaging metric alterations over between PLS patients and HC, a linear mixed effects model was implemented using R’s nmle package [32], where Time (i.e. session) was modelled as a random effect and the subjects as fixed effects. We corrected for the confounding effects of age, sex, handedness, and years of education. Volumetric analyses were also corrected for total cerebellar volumes (TCV) or total intracranial volumes (TIV) as appropriate, for cerebellar GM and cortical GM analyses respectively. We have evaluated the interaction effect “Time x Group” i.e., assessing if longitudinal progression was different in PLS compared to controls. Finally, we sought to compare whether disease burden was different in “cerebellar networks” compared to “primary motor networks”. First, we extracted the coefficients for the main effect “Group” (i.e., PLS vs. HC) from the ANOVA from all analysed ROIs and neuroimaging metrics (i.e., RD, FA, FC and volumetry), as described above. We then assigned, per neuroimaging metric, each ROI to the group “cerebellar network” or “primary motor network”. For example, for RD and FA, we compared the coefficients of the 11 assessed cerebellar tracts (i.e., bilateral cerebello-frontal/-parietal/-temporal/-occipital lobes/-thalamus and unified spinocerebellar) against the coefficients of three motor tracts (i.e., bilateral CST and transcallosal M1). To test for differential involvement of the two networks (supra- versus infratentorial), we used pairwise t-testing to test for means in the coefficients, regarding *p*-values < 0.05 as evidence of significant differences. Notice that for the volumetric analyses, *t*-testing was not possible since we only assessed volumetry for one ROI of the motor system (M1). Therefore, we provide instead descriptive statistics for the volumetric comparison. T-testing was performed within Matlab.

### Data availability

Due to departmental policies, clinical, genetic or neuroimaging data from individual patients cannot be made available, but additional information can be requested from the corresponding author with regard to statistics and data processing pipelines.

## Results

### Subjects

In total, data from 43 PLS patients and 113 HC were assessed. For most subjects, longitudinal data with up to three follow-up sessions were available. Multimodal MRI data included T1w, dMRI and rs-fMRI data, whereby not for all subjects/sessions, all sequences were available. We provide further details on available/missing data in Table 1. Demographic data were compared between PLS patients and HC: Welch two-sample t-testing indicated appropriate matching for age (t(80.33) = 1.33, *p* = 0.188), however, PLS patients had significantly fewer years of education t(71.04) =  – 4.05, *p* = 0.001). Chi-square tests revealed no differences in sex distributions between the groups (X2(1, *N* = 156) = 1.69, *p* = 0.193), and in distributions of handedness (X2(1, *N* = 156) = 0.11, *p* = 0.736). Patients with PLS tested negative for GGGGCC hexanucleotide expansions in *C9orf72* and the panel of HSP and ALS-associated genetic variants.

### Cross-sectional findings at baseline

**Fig. 2:**
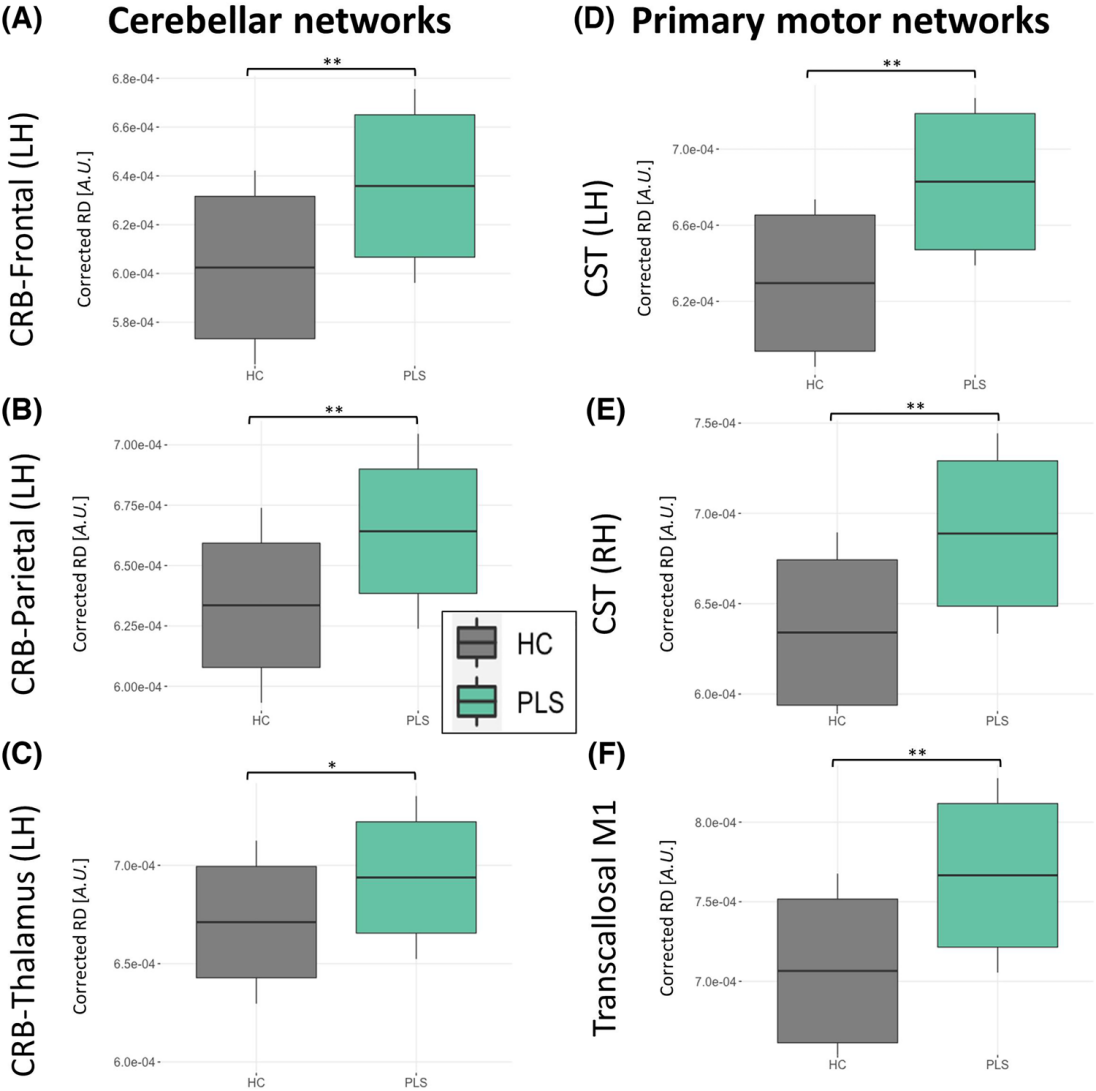
Structural connectivity (SC) profiles at baseline in cerebellar (**A**–**C**) and primary motor networks (**D**–**F**) *indicates *p*-values ≤ .05, **indicates p-values ≤ .001, *CRB* Cerebellar

**Fig. 3:**
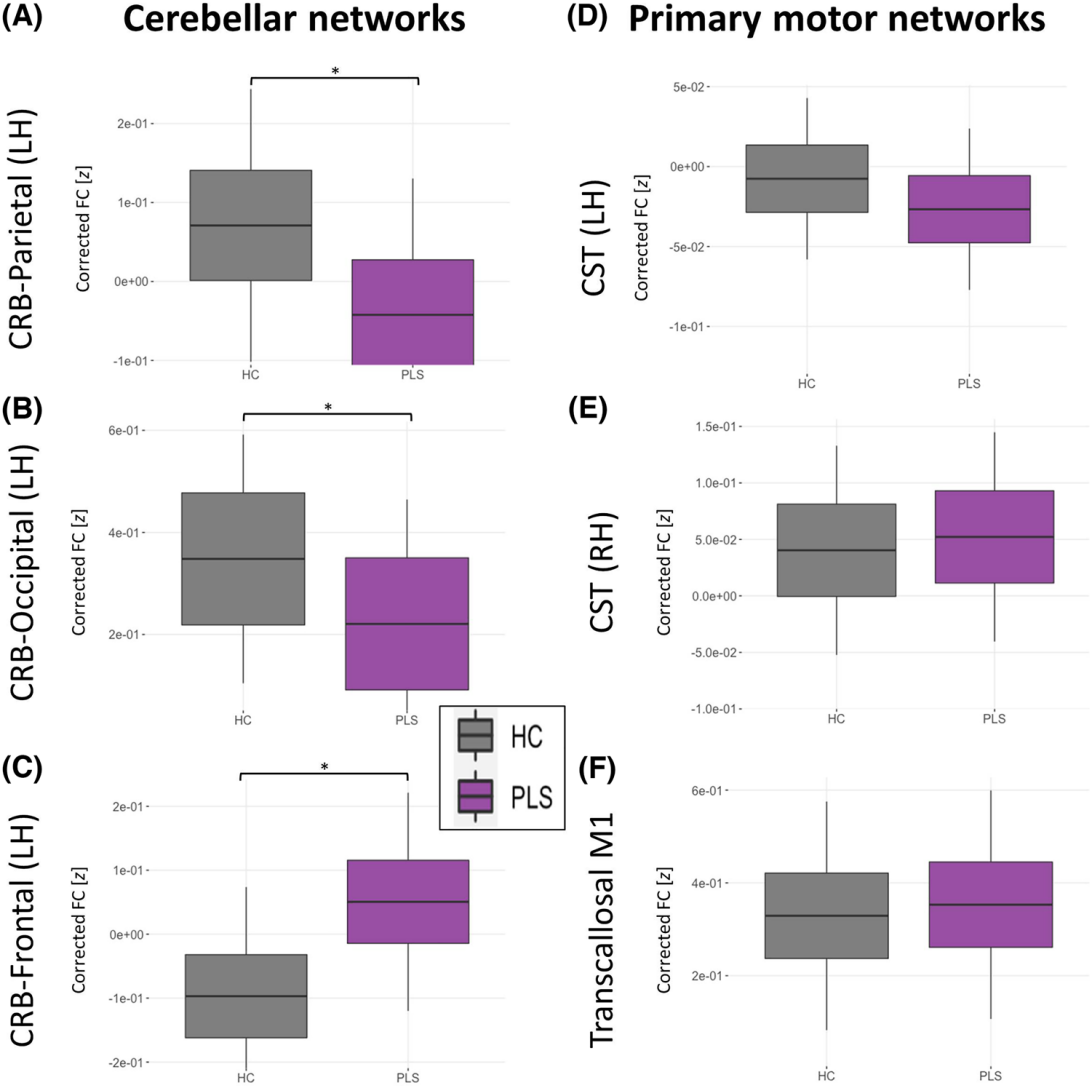
Functional connectivity (FC) profiles at baseline in cerebellar (**A**–**C**) and primary motor networks (**D**–**E**). *indicates *p*-values ≤ .05, **indicates *p*-values ≤ .001, *CRB* Cerebellar

**Fig. 4:**
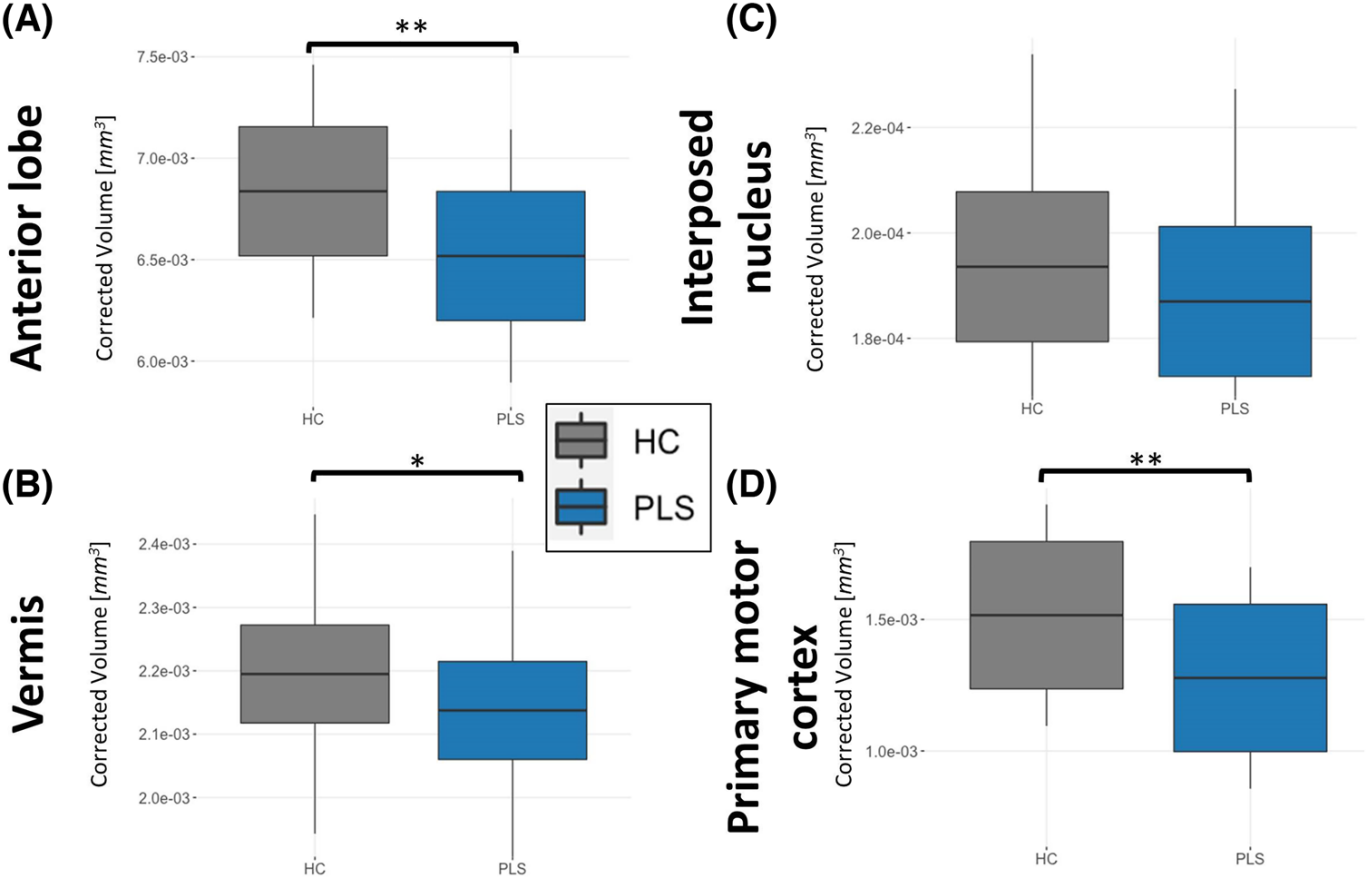
Volumetric profiles at baseline in cerebellar structures (**A**–**C**) and in the primary motor cortex (**D**) *indicates *p*-values ≤ .05, **indicates *p*-values ≤ .001

The PLS group exhibits infratentorial-supratentorial disconnection as compared to HC between the cerebellum and almost all lobes as well as the thalamus. This was found for both hemispheres and more evident for RD than FA. In Fig. 2, we present some illustrative RD findings between the cerebellum and frontal lobe (Fig. 2A), parietal lobe (Fig. 2B) and thalamus (Fig. 2C) tracts. With regards to the primary motor system, the CST (Fig. 2D–E) and trans-callosal tracts were also significantly affected (Fig. 2F) based on increased RD and decreased FA. Comprehensive descriptive statistics are provided in Table 2. and Fig. 3 highlights the key findings. The PLS groups display marked infratentorial/supratentorial disconnection, particularly between the cerebellum and parietal lobe (Fig. 3A) and the cerebellum and occipital lobes (Fig. 3B) in both hemispheres. Interestingly, the PLS group exhibits increased FC compared to HC between the cerebellum and LH frontal lobe (Fig. 3C). Unexpectedly, no FC differences were identified (Fig. 3D–F) in the primary motor system i.e. along the CST and CC. Further statistical details are provided in Table 2. Volume reductions were detected in PLS throughout the cerebellar cortex, including anterior (Fig. 4A) and posterior lobes, the vermis (Fig. 4B) and crura, but not in the flocculonodular lobe. We did not detect volumetric differences in the cerebellar nuclei, except for a tendency towards atrophy in the interposed nucleus (Fig. 4C). The motor cortex was significantly atrophic in PLS (Fig. 4D). Full statistics are provided in Table 2.

**Table 2:** Cross-sectional and longitudinal statistical comparisons of neuroimaging metrics between PLS and healthy controls

	Cross-sectional				Longitudinal (Interaction *Time x Group*)	
	Left hemisphere		Right hemisphere		Left hemisphere	Right hemisphere
	*F*-value (DOF)	*p*-value	*F*-value (DOF)	*p*-value	*t*-value (DOF), *p*-value	*t*-value (DOF), *p*-value
Connectivity: Crb-Frontal						
SC: RD	*F*(1,146) = 50.16	< 0.001**	*F*(1,146) = 56.89	< 0.001**	*t*(110) = 0.63, *p* = 0.530	*t*(110) = 1.30, *p* = 0.197
SC: FA	*F*(1,146) = 7.80	0.006*	*F*(1,146) = 4.25	00.041*	*t*(110) = – 0.92, *p* = 0.358	*t*(110) = – 1.93, *p* = .056
FC	*F*(1,142) = 6.80	0.010*	*F*(1,142) = .782	0.378	*t*(101) = – 1.16, *p* = 0.249	*t*(101) = – 0.01, *p* = 0.991
Connectivity: Crb-Parietal						
SC: RD	*F*(1,146) = 37.25	< 0.001**	*F*(1,146) = 36.28	< 0.001**	*t*(110) = 0.70, *p* = 0.485	*t*(110) = – 0.01, *p* = 0.988
SC: FA	*F*(1,146) = 2.22	0.138	*F*(1,146) = 1.47	0.228	*t*(110) = – 1.61, *p* = 0.111	*t*(110) = – 0.43, *p* = 0.666
FC	*F*(1,142) = 7.23	0.008*	*F*(1,142) = 17.41	< 0.001**	*t*(101) = 1.50, *p* = 0.138	*t*(101) = 2.25, *p* = 0.027*
Connectivity: Crb-Temporal						
SC: RD	*F*(1,146) = 10.44	0.001**	*F*(1,146) = 25.68	< 0.001**	*t*(110) = 1.01, *p* = 0.315	*t*(110) = 0.05, *p* = 0.958
SC: FA	*F*(1,146) = 2.87	0.092	*F*(1,146) = 3.09	0.081	*t*(109) = – 0.66, *p* = 0.508	*t*(109) = 0.18, *p* = 0.859
FC	*F*(1,142) = 0.11	0.738	*F*(1,142) = 0.20	0.654	*t*(101) = – 1.75, *p* = 0.084	*t*(101) = – 0.72, *p* = 0.473
Connectivity: Crb-Occipital						
SC: RD	*F*(1,146) = 16.40	< 0.001**	*F*(1,146) = 31.84	< 0.001**	*t*(110) = 0.16, *p* = 0.873	*t*(110) = – 0.99, *p* = 0.326
SC: FA	*F*(1,146) = 0.30	0.583	*F*(1,146) = 0.007	0.933	*t*(110) = 0.17, *p* = 0.864	*t*(110) = 0.62, *p* = 0.534
FC	*F*(1,142) = 6.49	0.012*	*F*(1,142) = 3.861	0.051	*t*(101) = – 1.53, *p* = 0.130	*t*(101) = 0.29, *p* = 0.772
Connectivity: Crb-Thalamus						
SC: RD	*F*(1,146) = 5.21	0.024*	*F*(1,146) = 3.44	0.066	*t*(110) = 0.19, *p* = 0.851	*t*(110) = 0.44, *p* = 0.658
SC: FA	*F*(1,146) = 0.12	0.728	*F*(1,146) = 0.07	0.790	*t*(110) = – 0.06, *p* = 0.955	*t*(110) = 0.47, *p* = 0.637
FC	*F*(1,142) = 0.33	0.566	*F*(1,142) = 2.45	0.120	*t*(101) = 0.44, *p* = 0.662	*t*(101) = 0.84, *p* = 0.404
Connectivity: Spinocerebellar						
SC: RD	*F*(1,146) = 2.26	0.135	n.a	n.a	*t*(110) = – 0.30, *p* = 0.764	n.a
SC: FA	*F*(1,146) = 1.18	0.279	n.a	n.a	*t*(110) = – 0.65, *p* = 0.515	n.a
FC	*F*(1,142) = 0.33	0.566	n.a	n.a	*t*(101) = 0.15, *p* = 0.882	n.a
Connectivity: Corticospinal tract						
SC: RD	*F*(1,146) = 50.30	< 0.001**	*F*(1,146) = 50.41	< 0.001**	*t*(110) = 1.06, *p* = 0.289	*t*(110) = 1.57, *p* = 0.119
SC: FA	*F*(1,146) = 27.64	< 0.001**	*F*(1,146) = 24.96	< 0.001**	*t*(110) = 1.06, *p* = 0.289	*t*(110) = – 1.74, *p* = 0.084
SC: FC	*F*(1,142) = 0.28	0.596	*F*(1,142) = 0.32	0.571	*t*(101) = 1.57, *p* = 0.121	*t*(101) = 0.04, *p* = 0.970
Connectivity: Transcallosal M1 (hemisphere n.a.)						
SC: RD	*F*(1,146) = 39.10	< 0.001**	n.a	n.a	*t*(110) = 1.85, *p* = 0.066	n.a
SC: FA	*F*(1,146) = 14.76	0.002**	n.a	n.a	*t*(110) = – 1.05, *p* = 0.297	n.a
FC	*F*(1,142) = 0.63	0.427	n.a	n.a	*t*(101) = – 0.96, *p* = 0.340	n.a
Volumetry: Cerebellar cortex (hemisphere: n.a.)						
Anterior cerebellar lobe	*F*(1,146) = 11.37	< 0.001**	n.a	n.a	*t*(112) = – 1.11, *p* = 0.270	n.a
Posterior cerebellar lobe	*F*(1,146) = 9.18	0.002*	n.a	n.a	*t*(112) = – 1.27, *p* = 0.208	n.a
Flocculonodular lobe	*F*(1,146) = 1.04	0.309	n.a	n.a	*t*(112) = – 1.85, *p* = 0.067	n.a
Cerebellar crura	*F*(1,146) = 5.02	0.027*	n.a	n.a	*t*(112) = – 1.53, *p* = 0.128	n.a
Cerebellar vermis	*F*(1,146) = 5.51	0.020*	n.a	n.a	*t*(112) = – 1.01, *p* = 0.314	n.a
Volumetry: Cerebellar nuclei (hemisphere: n.a.)						
Dorsal dentate nucleus	*F*(1,146) = 5.02	0.027*	n.a	n.a	*t*(112) = 0.87, *p* = 0.384	n.a
Ventral dentate nucleus	*F*(1,146) = 1.21	0.274	n.a	n.a	*t*(112) = 0.94, *p* = 0.348	n.a
Interposed nucleus	*F*(1,146) = 3.80	0.053	n.a	n.a	*t*(112) = 0.78, *p* = .435	n.a
Fastigial nucleus	*F*(1,146) = 0.41	0.521	n.a	n.a	*t*(112) = 0.48, *p* = 0.636	n.a
Volumetry: Motor cortex (hemisphere: n.a.)						
M1	*F*(1,146) = 58.36	< 0.001**	n.a	n.a	*t*(112) = – 1.01, *p* = 0.316	n.a

*ANOVA* analysis of variance, *Crb* cerebellum, *dMRI* diffusion-magnetic resonance imaging, *DOF* degrees of freedom, *FA* fractional anisotropy, *FC* functional connectivity, *HC* healthy control, *M1* primary motor cortex, *n.a.* not applicable, *RD* radial diffusivity, *PLS* primary lateral sclerosis, *rs-fMRI* resting-state functional MRI, *SC* structural connectivity, *T1w* T1-weighted MRI

^*^significant at an alpha-level of *p* ≤ 0.05

^**^significant at an alpha-level of *p* ≤ 0.001

### Longitudinal analyses

**Fig. 5:**
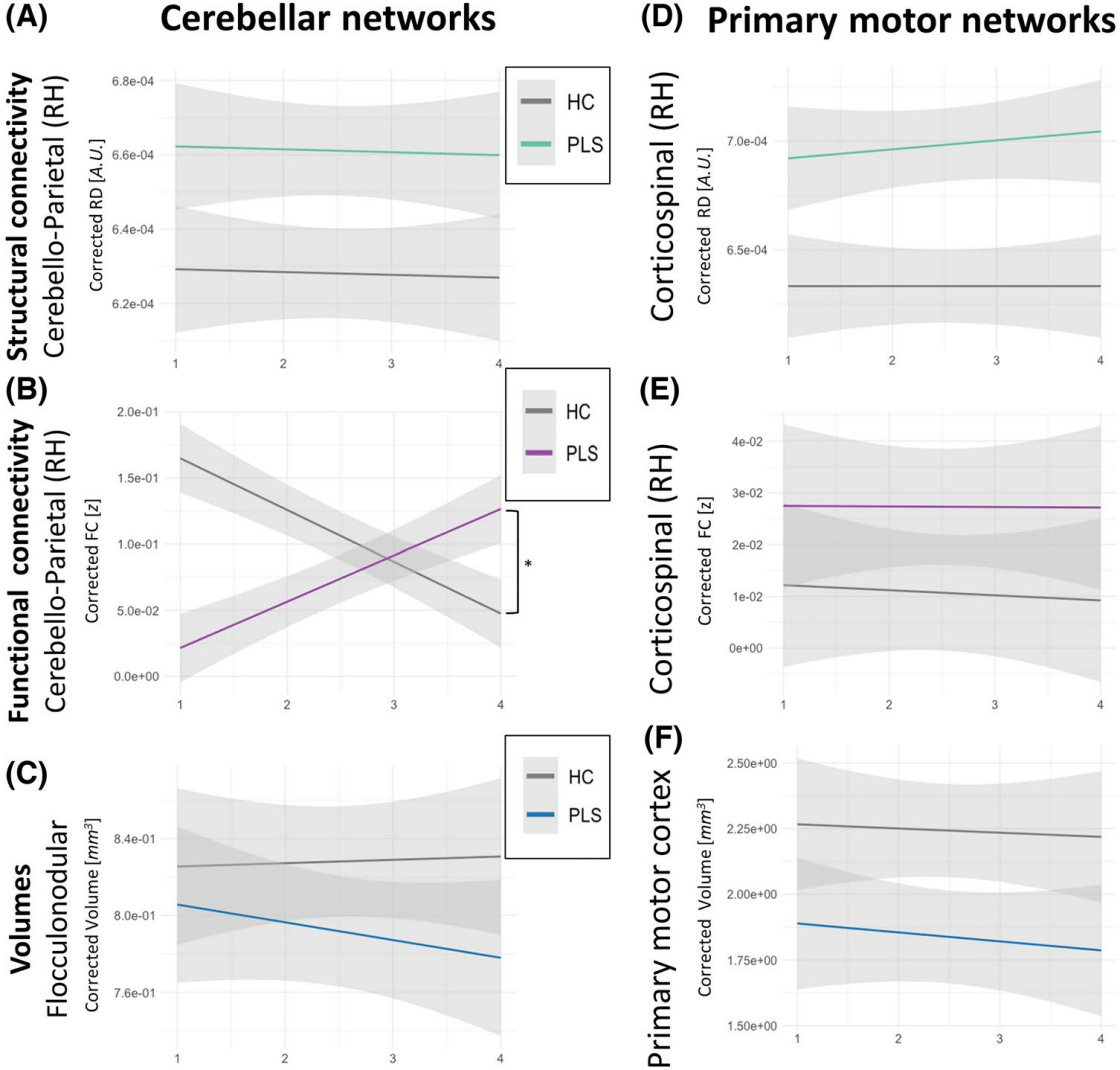
The longitudinal evolution of cerebral and cerebellar integrity metrics. *indicates *p*-values ≤ .05, **indicates *p*-values ≤ .001

With regards to longitudinal structural connectivity, we found no evidence for divergent longitudinal progression between PLS and HC in any of the cerebellar (e.g., Fig. 5A for RD Cerebellum-to-parietal lobe, RH) or primary motor tracts (e.g., Fig. 5D for RD CST, RH). The results were similar for RD and FA. To assess differences in the evolution of FC, a linear mixed effects model was implemented to assess the interaction effect “Time x Group”, correcting for age, sex, handedness, and years of education. We found no evidence of accelerated FC disconnection in PLS compared to HC in any of the analyzed networks in cerebellar or primary motor circuits (Fig. 5E for FC of CST, RH). However, we detected a marked increase in FC in PLS over time between the cerebellum and RH parietal lobe (Fig. 5B). Differences in longitudinal volumetric trajectories between PLS and HC were also evaluated, but no significant differences were detected, neither for the cerebellar cortex (except for a tendency of accelerated atrophy of the flocculonodular lobe, Fig. 5C), cerebellar nuclei (not shown) or the motor cortex (e.g., Fig. 5F).

### Divergent disease burden in the primary motor system and cerebellar networks

**Fig. 6:**
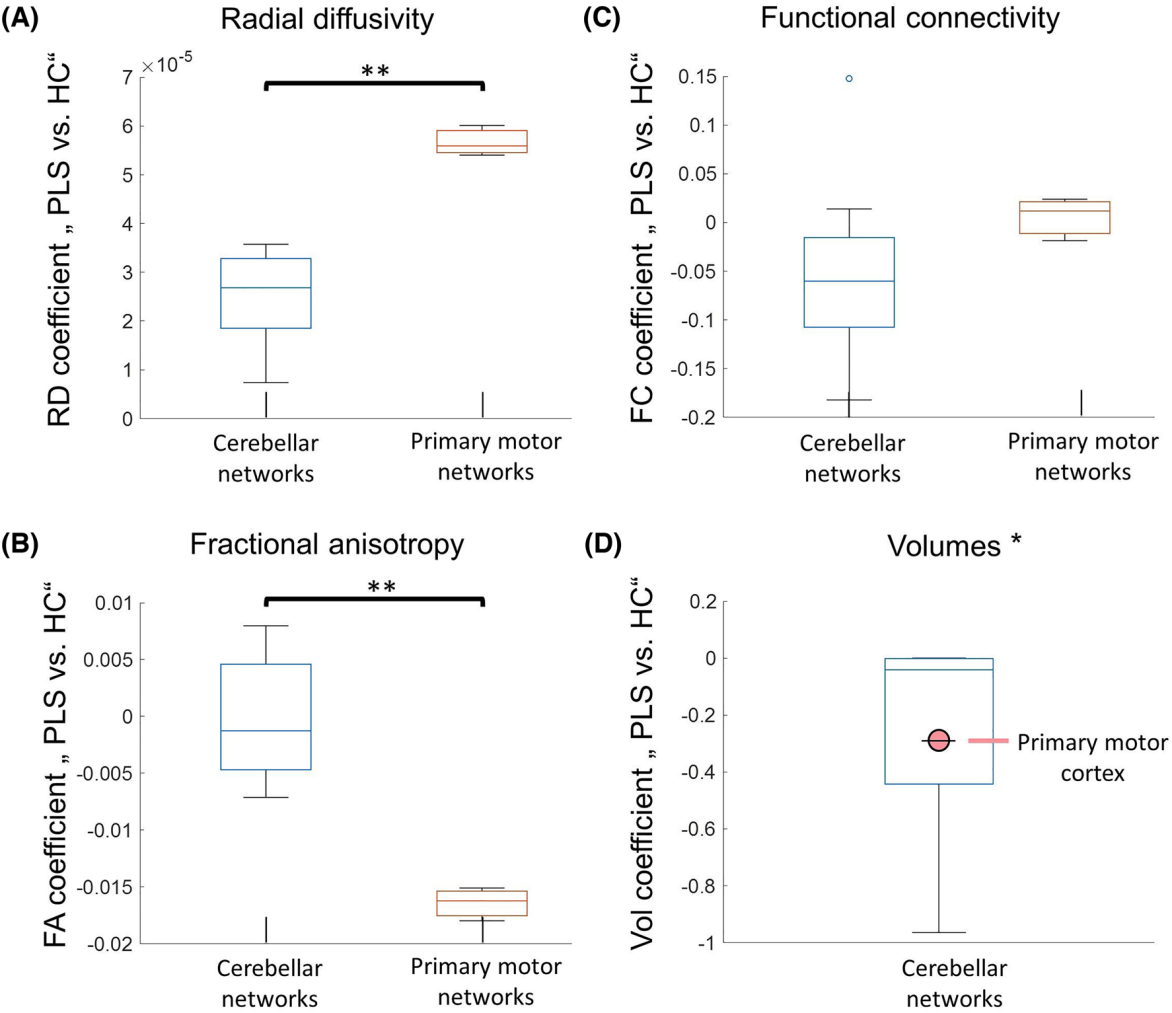
The comparison of cerebral and cerebellar disease burden at baseline. *indicates *p*-values ≤ .05, **indicates *p*-values ≤ .001

To test for disease burden differences between cerebellar and primary motor region, pairwise t-testing was used, comparing means of the coefficient of the main effect “Group” from all ROIs in each system. This analysis was repeated for each neuroimaging metric (Fig. 6). Baseline disease burden was more pronounced in the motor system as compared to the cerebellar system for SC, as evident by higher RD coefficients (Fig. 6A, t(12) =  – 5.78, *p* < 0.001) and lower FA coefficients (Fig. 6B, t(12) = 5.16, p < 0.001), but not for FC (Fig. 6C, t(12) =  – 1.05, *p* = 0.312). For the volumetric analysis (Fig. 6D), t-testing was not possible since only one value was available for the motor system. The mean/standard deviation of the volumetric coefficients of the assessed cerebellar ROIs was -0.258 ± 0.359, the coefficient for M1 volumetry was – 0.348.

## Discussion

Our data confirms considerable intra-cerebellar disease burden in PLS. We detected cerebello-frontal, temporal, parietal, occipital and cerebello-thalamic structural disconnection at baseline as well as impaired cerebello-frontal, -parietal and -occipital functional connectivity. Volume reductions were identified in the vermis, anterior lobe, posterior lobe, and crura. Among the deep cerebellar nuclei, the dorsal dentate was atrophic. Interestingly longitudinal follow-up did not capture statistically significant progressive changes. Consistent with classical imaging signatures, significant primary motor cortex atrophy and inter-hemispheric transcallosal degeneration was also captured based on diffusivity metrics but not based on functional analyses.

### Academic insights

Cortical cerebellar changes have been previously described in PLS, and our data also suggest widespread cerebellar grey matter change including the vermis, anterior lobe, posterior lobe, and crura. Posterior cerebellar pathology has been consistently linked to visuospatial, language, verbal memory, executive and sequencing deficits; while neuropsychological function may be relatively preserved with anterior cerebellar insults [68]. Cerebellar disease burden has also been linked to deficits in social cognition [69], language [70] and pseudobulbar affect [17, 20]. Vermis lesions may manifest emotional dysregulation such as impulsivity, irritability, and disinhibition [71]. Previous imaging studies have reported cerebellar [9], spinocerebellar and dentato-rubro-thalamo-cortical (DRTC) tract diffusivity changes [72] as well as increased functional connectivity between the cerebellum and cortical motor, frontal and temporal areas [73]. Cognitive deficits in PLS have been linked to reduced FA and higher cerebellar RD [74]. Increased cerebro-cerebellar functional connectivity and expanded metabolic activation are also commonly reported in ALS [75, 76] and often interpreted as functional adaptation to neurodegenerative change [77, 78]. While concepts of neuroplasticity and compensatory mechanisms are attractive at a theoretical level, there is no compelling histopathology evidence to convincingly support adaptive structural processes [79].

### Methodological considerations

From a methodological perspective, our results highlight the relative limitations of functional connectivity metrics. In our study, the detection sensitivity of structural connectivity indices clearly outperforms BOLD-signal-derived functional measures. This is potentially important for the design of short-duration clinical protocols, which should ideally only include pulse sequences with biomarker potential. While FA is the most widely evaluated diffusivity metric both in tractography and voxelwise models such as TBSS etc., our study highlights the importance of assessing other measures. In this study, RD was more sensitive to detect cortico-cerebellar disconnection than FA. It is noteworthy, that we did not detect significant longitudinal structural or functional changes, which is likely to stem from the long symptom duration profile of our sample. It is particularly striking that corticospinal tract, corpus callosum and primary motor cortex measures did not exhibit progressive decline either, despite the relentless clinical progression observed clinically. This suggests a saturation-effect or ceiling effect of these variables i.e. while they capture significant differences with reference to control change there is no tangible further progression over time. One needs to also acknowledge the heterogeneity of the cohort with respect to symptom duration as well as the long average length of symptom duration. By the time these participants have been scanned they have had the disease for a long time, resulting in considerable CST, CC, and PMC degeneration which has not progressed further significantly during the relatively short 4-monthly follow-up periods. Accordingly, it would be particularly interesting to capture patients soon after symptom onset, before fulfilling diagnostic criteria or patients with “suspected” disease and evaluate their imaging change at a much earlier stage of the disease. We have made such attempts previously, evaluating patients with a symptom duration of 2–4 years i.e. patients with “probable PLS” according to the new consensus criteria [27] and they have already exhibited motor cortex and corticospinal tract alterations [28, 80]. Imaging patients with short symptom duration is particularly important as these are the patients who face diagnostic uncertainty, apprehensive about potentially developing ALS [81]. In this study, we used a single inferior (caudal) brainstem mask to track ascending projections to the cerebellum as a proxy of the “spinocerebellar tracts”, but we concede that this is a suboptimal approach. High-resolution spinal data can now be reliably acquired with cardiac and respiratory gating in ALS [82] and correlated with clinical metrics [83, 84]. Spinal protocols in ALS have also been successfully utilised in diagnostic applications [85] and to describe presymptomatic changes in *C9orf72* [86]. Combined spinal and cerebral datasets are increasingly evaluated in ALS to accurately explore the substrate of clinical phenomena [83].

### Clinical implications

From a pathophysiology standpoint, functional MRI studies in ALS [75, 77, 79] have consistently suggested that the cerebellum may assume a compensatory role in the face of supratentorial degeneration and cerebellar hypertrophy has also been detected in adult poliomyelitis survivors [87, 88]. In our study, PLS patients exhibit increased functional connectivity compared to controls between the cerebellum and left frontal lobe at baseline (Fig. 3C) and increasing cerebello-parietal FC over time (Fig. 5B). As the increased FC is not supported by increased SC, one needs to be careful not to over-interpret BOLD synchronisation as proof of effective compensation. Our volumetric analyses revealed a trend for progressive flocculonodular lobe atrophy in PLS (Fig. 5). This region is a key hub of the vestibulo-ocular networks playing an important role in fixation, smooth pursuit, and vestibular responses to head motion. These observations support the importance of eye-movement assessments in PLS, particularly that other cerebellar manifestations, such as disequilibrium during stance and gait are affected by coexisting pyramidal weakness or spasticity. While eye-movement abnormalities have been extensively investigated in ALS [43], they are glaringly understudied in PLS. Cerebellar atrophy and cortico-cerebellar connectivity changes are also commonly observed in ALS, ALS-FTD and HSP [89–91]. Radiological measures are increasingly included as input features in machine-learning (ML) models in MNDs [92, 93], but these have only achieved limited accuracy when distinguishing ALS from PLS on radiological grounds [94]. The development of accurate classification models that can successfully differentiate ALS from PLS soon after symptom onset is of utmost relevance given the strikingly different survival prospects in the two conditions [95]. From a clinical perspective, the specific contribution of cerebellar pathology to dexterity and gait impairment, dysarthria and dysphagia is difficult to demonstrate in the presence of considerable upper motor neuron dysfunction. Based on similar research studies in ALS, [76, 96], it is likely that extra-pyramidal [13] and cerebellar pathology in PLS [32] exacerbates motor disability in PLS. Accordingly, cerebellar components of motor disability should be considered in fall prevention, multidisciplinary rehabilitation, occupational, speech and physiotherapy.

### Study limitations

This study is not without limitations. We acknowledge the relative heterogeneity of our sample with regards to symptom duration and that only 31 of the 43 patients had genomic data. We also concede that supplementary spectroscopy and PET data would have provided important complementary metabolic insights. Additional spinal MRI data would have permitted the direct evaluation of the spinocerebellar tracts instead of relying on an inferior brainstem mask for tractography. Notwithstanding these limitations, our data demonstrate considerable intra-cerebellar and well as significant cerebro-cerebellar connectivity alterations in PLS.

## Conclusions

PLS is associated with considerable cerebellar disease burden; cortical atrophy, dorsal dentate degeneration and cerebro-cerebellar connectivity alterations. Cerebellar components of gait impairment, bulbar dysfunction and pseudobulbar affect in PLS should be carefully considered instead of attributing characteristic symptoms to primary motor cortex degeneration alone.

### Supplementary Information

Below is the link to the electronic supplementary material.

Supplementary file1 (DOCX 21 KB)

## Abbreviations

AAL: Automated Anatomical Labeling (AAL) atlas
AD: Alzheimer’s disease
ALS: Amyotrophic lateral sclerosis
ALSod: ALS online database
ANOVA: Analysis of variance (ANOVA)
ASO: Antisense oligonucleotide
BOLD: Blood-oxygen-level-dependent (BOLD) signal
C9 +: ALS patients with GGGGCC hexanucleotide repeat expansion in C9orf72
C9-: ALS patients without GGGGCC hexanucleotide repeat expansion in C9orf72
*C9orf72*: Chromosome 9 open reading frame 72
CC: Corpus callosum
CNS-LS: Center for Neurological Study- Lability Scale
CSD: Constrained spherical deconvolution
CST: Corticospinal tract
CT: Cortical thickness
DOFs: Degrees of freedom
dMRI: Diffusion magnetic resonance imaging
ECAS: Edinburgh Cognitive and Behavioural ALS Screen
ELQ: Emotional Lability Questionnaire
EMG: Electromyography
EMM: Estimated marginal mean
EPI: Echo-planar imaging
FA: Fractional anisotropy
FC: Functional connectivity
fMRI: Functional MRI
FLAIR: Fluid-attenuated inversion recovery
fODF: Fibre orientation distribution function
FOV: Field of view
FrSBe: Frontal Systems Behavior Scale
FSL: FMRIB´s Software Library
FTD: Frontotemporal dementia
FTLD: Frontotemporal lobar degeneration
FWE: Familywise error
GM: Gray matter
HADS: Hospital Anxiety and Depression Scale
HARDI: High Angular Resolution Diffusion Imaging
HC: Healthy control
HD: Huntington’s disease
ICA-AROMA: Automatic Removal Of Motion Artifacts
IR-SPGR: Inversion recovery prepared spoiled gradient recalled echo
IQR: Interquartile range
LAS: Local Adaptive Segmentation
LH: Left hemisphere
Lt: Left
LMN: Lower motor neuron
M1: Primary motor cortex
ML: Machine-learning
MND: Motor neuron disease
MNI: Montreal Neurological Institute
MNI152: Montreal Neurological Institute 152 standard space
MRI: Magnetic resonance imaging
MRS: MR spectroscopy
NISALS: Neuroimaging Society in ALS
NIV: Non-invasive ventilation
NODDI: Neurite orientation dispersion and density imaging
PBA: Pseudobulbar affect
PCR: Polymerase chain reaction
PD: Parkinson’s disease
PMC: Primary motor cortex
PUMNS: Penn Upper Motor Neuron Score
QC: Quality control
RH: Right hemisphere
Rt: Right
RD: Radial diffusivity
ROI: Region of interest
rs-fMRI: Resting-state functional MRI
SC: Structural connectivity
SD: Standard deviation
SE-EPI: Spin echo planar imaging
SENSE: Sensitivity Encoding
SPIR: Spectral presaturation with inversion recovery
T: Tesla
T1w: T1-weighted imaging
TCV: Total cerebellar volume
TDI: Track Density Imaging
TE: Echo time
TI: Inversion time
TIV: Total intracranial volume
Tukey HSD: Tukey’s Honest Significant Difference
TR: Repetition time
UMN: Upper motor neuron
VR: Voxel resolution
WM: White matter
