# Supplementary material for: Supra- and infra-tentorial degeneration patterns in primary lateral sclerosis: a multimodal longitudinal neuroradiology study
Source: J Neurol. 2024 Mar 5;271(6):3239–55. doi: 10.1007/s00415-024-12261-z (PMC11136747; doi:10.1007/s00415-024-12261-z)
Supplement: Supplementary file 1 — Supplementary file1 (DOCX 21 KB) [file 415_2024_12261_MOESM1_ESM.docx]

**STROBE STATEMENT**

**Title:** Supra- and infra-tentorial degeneration patterns in Primary Lateral Sclerosis: a multimodal longitudinal neuroradiology study

**STROBE statement**

The presented pilot observational study complies with the STROBE statement guidelines. The requirements of all Items (1-22) on the bellow STROBE checklist were met.

|  | **Item No** | **Recommendation** |
| --- | --- | --- |
| **Title and abstract** | 1 **✓** | (*a*) Indicate the study’s design with a commonly used term in the title or the abstract |
|  |  | (*b*) Provide in the abstract an informative and balanced summary of what was done and what was found |
| **Introduction** | | |
| Background/rationale | 2 **✓** | Explain the scientific background and rationale for the investigation being reported |
| Objectives | 3 **✓** | State specific objectives, including any prespecified hypotheses |
| **Methods** | | |
| Study design | 4 **✓** | Present key elements of study design early in the paper |
| Setting | 5 **✓** | Describe the setting, locations, and relevant dates, including periods of recruitment, exposure, follow-up, and data collection |
| Participants | 6 **✓** | (*a*) Give the eligibility criteria, and the sources and methods of case ascertainment and control selection. Give the rationale for the choice of cases and controls |
|  |  | (*b*) For matched studies, give matching criteria and the number of controls per case |
| Variables | 7 **✓** | Clearly define all outcomes, exposures, predictors, potential confounders, and effect modifiers. Give diagnostic criteria, if applicable |
| Data sources/ measurement | 8* **✓** | For each variable of interest, give sources of data and details of methods of assessment (measurement). Describe comparability of assessment methods if there is more than one group |
| Bias | 9 **✓** | Describe any efforts to address potential sources of bias |
| Study size | 10 **✓** | Explain how the study size was arrived at |
| Quantitative variables | 11 **✓** | Explain how quantitative variables were handled in the analyses. If applicable, describe which groupings were chosen and why |
| Statistical methods | 12 **✓** | (*a*) Describe all statistical methods, including those used to control for confounding |
|  |  | (*b*) Describe any methods used to examine subgroups and interactions |
|  |  | (*c*) Explain how missing data were addressed |
|  |  | (*d*) If applicable, explain how matching of cases and controls was addressed |
|  |  | (*e*) Describe any sensitivity analyses |
| **Results** | | |
| Participants | 13* **✓** | (a) Report numbers of individuals at each stage of study—eg numbers potentially eligible, examined for eligibility, confirmed eligible, included in the study, completing follow-up, and analysed |
|  |  | (b) Give reasons for non-participation at each stage |
|  |  | (c) Consider use of a flow diagram |
| Descriptive data | 14* **✓** | (a) Give characteristics of study participants (eg demographic, clinical, social) and information on exposures and potential confounders |
|  |  | (b) Indicate number of participants with missing data for each variable of interest |
| Outcome data | 15* **✓** | Report numbers in each exposure category, or summary measures of exposure |
| Main results | 16 **✓** | (*a*) Give unadjusted estimates and, if applicable, confounder-adjusted estimates and their precision (eg, 95% confidence interval). Make clear which confounders were adjusted for and why they were included |
|  |  | (*b*) Report category boundaries when continuous variables were categorized |
|  |  | (*c*) If relevant, consider translating estimates of relative risk into absolute risk for a meaningful time period |
| Other analyses | 17 **✓** | Report other analyses done—eg analyses of subgroups and interactions, and sensitivity analyses |
| **Discussion** | | |
| Key results | 18 **✓** | Summarise key results with reference to study objectives |
| Limitations | 19 **✓** | Discuss limitations of the study, taking into account sources of potential bias or imprecision. Discuss both direction and magnitude of any potential bias |
| Interpretation | 20 **✓** | Give a cautious overall interpretation of results considering objectives, limitations, multiplicity of analyses, results from similar studies, and other relevant evidence |
| Generalisability | 21 **✓** | Discuss the generalisability (external validity) of the study results |
| **Other information** | | |
| Funding | 22 **✓** | Give the source of funding and the role of the funders for the present study and, if applicable, for the original study on which the present article is based |

I confirm that the above STROBE guideline recommendations were met.

Peter Bede MD PhD

Trinity College Dublin

21/12/2023
